# Association of Physician Group Practice Participation in Bundled Payments With Patient Selection, Costs, and Outcomes for Joint Replacement

**DOI:** 10.1001/jamahealthforum.2021.0295

**Published:** 2021-05-06

**Authors:** Karen E. Joynt Maddox, E. John Orav, Jie Zheng, Arnold M. Epstein

**Affiliations:** 1 Cardiovascular Division, Department of Medicine, Washington University School of Medicine in St Louis, St Louis, Missouri; 2 Center for Health Economics and Policy, Institute for Public Health, Washington University in St Louis, St Louis, Missouri; 3 Department of Biostatistics, Harvard T.H. Chan School of Public Health, Boston, Massachusetts; 4 Division of General Internal Medicine, Department of Medicine, Brigham and Women’s Hospital, Boston, Massachusetts; 5 Department of Health Policy and Management, Harvard T.H. Chan School of Public Health, Boston, Massachusetts

## Abstract

**Importance:**

Medicare’s Bundled Payments for Care Improvement (BPCI) program, which ran from 2013 to 2018, was an important experiment in physician-focused alternative payment models. However, little is known about whether the program was associated with better quality or outcomes or lower costs.

**Objective:**

To determine whether participation in BPCI among physician group practices was associated with advantageous or deleterious changes in costs or patient outcomes.

**Design, Setting, and Participants:**

This cross-sectional study used 2013 to 2017 Medicare files and difference-in-differences (DID) models to compare the change over time in Medicare payments, patient selection, and clinical outcomes between 91 orthopedic groups in BPCI Model 2 and 169 propensity-matched controls for patients undergoing joint replacement. Analyses were performed between December 2019 and February 2021.

**Exposures:**

Voluntary participation in BPCI.

**Main Outcomes and Measures:**

The primary outcome was 90-day Medicare payments; secondary outcomes were patient selection (volume, comorbidities) and clinical outcomes (30-day and 90-day emergency department visits, readmissions, mortality, and healthy days at home).

**Results:**

There were 74 343 patient episodes in the baseline period and 102 790 during the intervention in BPCI practices, and 88 147 patient episodes in the baseline period and 120 253 during the intervention in control practices; 291 214 of 461 598 (63.1%) patients were women, and 419 619 (90.9%) were White. At baseline, mean episode payments among BPCI-participating practices were $18 257, which decreased to $15 320 during the intervention, while control practices decreased from $17 927 to $16 170 (DID, −$1180; 95% CI, −$1565 to −$795; *P* < .001). Savings were driven by a decrease in postacute care spending. There were no differential changes in volume or comorbidities. The BPCI practices increased the proportion of patients discharged home compared with controls (23.6% to 43.4% vs 22.2% to 31.8%; DID, 10.2% [95% CI, 6.2% to 14.1%]). There were no differential changes in 30-day or 90-day mortality rates or emergency department visits, but 30-day and 90-day readmission rates decreased more among BPCI practices than controls (90 days: 8.7% to 7.5% vs 8.9% to 8.7%; DID, −1.0% [95% CI, −1.4% to −0.5%]), and 90-day healthy days at home increased (BPCI, 82.9 to 84.8, vs controls, 83.1 to 84.4; DID, 0.6 [95% CI, 0.4 to 0.8]).

**Conclusions and Relevance:**

Group practice participation in BPCI for joint replacement was associated with reduced Medicare payments and improvements in clinical outcomes.

## Key Points

**Question:**

Was participation in the Bundled Payments for Care Improvement (BPCI) initiative among physician group practices associated with changes in Medicare payments, patient selection, or clinical outcomes?

**Findings:**

In this cross-sectional study of 91 orthopedic groups in BPCI Model 2 and 169 propensity-matched controls, BPCI-participating practices decreased 90-day Medicare payments from $18 257 to $15 320 during the intervention, while control practices decreased payments from $17 927 to $16 170; 30-day and 90-day readmission rates decreased more among BPCI practices than controls, and 90-day healthy days at home increased.

**Meaning:**

Group practice participation in BPCI for joint replacement was associated with reduced Medicare payments and improvements in clinical outcomes.

## Teaser

This cross-sectional study assesses whether physician group participation in Medicare bundled payments is associated with changes in costs or patient outcomes.

## Introduction

Medicare and other payers are increasingly moving toward alternative payment models in which clinicians are paid for the quality, rather than solely for the quantity, of care they provide. One example is the Bundled Payments for Care Improvement (BPCI) program, launched by Medicare in 2013. Hospitals, postacute care facilities, and physician group practices (PGPs) that joined assumed responsibility for quality and costs of care for a 30-day, 60-day, or 90-day episode that started with a hospital admission for 1 of 48 medical or surgical conditions.

Perhaps the most successful area for BPCI has been for major joint replacement of the lower extremity (MJRLE). Among hospital and postacute facility participants, studies have shown lower Medicare payments compared with concurrent controls,^1,2,3^ though according to a recent publication from the Center for Medicare & Medicaid Innovation,^4^ those improvements did not lead to savings for Medicare because they were offset by bonus payments paid out under the program. However, despite the fact that BPCI represented a large, national experiment in alternative payment mechanisms and enrolled hundreds of physician groups, little is known about whether the program was associated with better quality or outcomes or lower costs.

Physician group practices could fare differently under BPCI than hospitals or postacute facilities. They may be more nimble in changing postdischarge follow-up to prevent rehospitalizations, and they may have preexisting relationships with patients that help them determine cost-effective postacute care pathways. Additionally, because their revenue is driven by professional fees rather than facility fees, they may benefit less from rehospitalizations. Studies of the largest alternative payment model under Medicare, the Medicare Shared Savings Program, showed that accountable care organizations led by PGPs were more likely to succeed.^5^

As the Centers for Medicare & Medicaid Services (CMS) and other payers continue to seek payment models that reduce costs and improve quality, understanding the association of PGP participation in BPCI with costs and outcomes is important. Furthermore, in October 2018, Medicare launched a follow-on program to BPCI, called BPCI-Advanced, that was joined by more than 500 PGPs^6^ but was subsequently put on hold during the COVID-19 pandemic. Understanding patterns in BPCI could be important to informing program design and monitoring strategies under BPCI-Advanced.

In this study, we aimed to fill this gap by examining changes in payments, patient selection, and clinical outcomes for patients of PGPs participating in BPCI. We focused on MJRLE because it is the most common type of surgery performed among Medicare beneficiaries^7^ and was the most commonly selected condition by PGP participants in BPCI.

## Methods

### Overview of BPCI

There were 4 participation models in BPCI, but 79% of PGPs enrolled in Model 2,^8^ in which participants assumed accountability for all Medicare payments (eg, initial hospitalization, readmission, postacute care) during an episode triggered by a hospital admission and continuing for 30, 60, or 90 days postdischarge. The PGPs were paid fee-for-service rates, but payments were retrospectively reconciled against targets on a quarterly basis. Target prices were set based on each practice’s unique historical utilization, minus a discount of 2% to 3% depending on the episode length chosen by the participating entity.

This study was approved by the Office of Human Research Administration at the Harvard School of Public Health. The requirement for informed consent was waived because the data were deidentified. This study followed the Strengthening the Reporting of Observational Studies in Epidemiology (STROBE) reporting guideline.

### BPCI Participants and Controls

More information on this process is provided in the eMethods in the Supplement. In brief, we obtained public data from Medicare listing BPCI participants along with their start dates and the date they terminated participation. We merged this list with the Medicare Data on Provider Practice and Specialty data set. We limited the sample to orthopedic surgery practices (which comprised 93 of 96 participants with at least 1 MJRLE performed during both the preperiods and postperiods) for both participants and controls to enable more appropriate comparisons.

We identified the National Provider Identifier (NPI) numbers for physicians affiliated with each practice in each year. When NPIs were affiliated with more than 1 taxpayer identification number (TIN) (19.6% of all NPIs are billed under 2 or more TINs), we used data from the Medicare Carrier file to assign them to the TIN with which they billed the plurality of their claims for MJRLE. For both BPCI and potential control TINs, we linked these data at the TIN level to public data from Physician Compare for practice characteristics, such as location and size, and at the county level to the Area Resource File for market characteristics, such as median income. Market share was calculated as the proportion of all admissions in a county for MJRLE by each PGP. Market competitiveness was calculated using the Herfindahl-Hirschman Index.^9^

To select controls, we used a modification of an approach used in a prior study of BPCI.^1^ Using propensity scores based on PGP and market characteristics, each BPCI PGP was matched without replacement with up to 3 control orthopedic PGPs within the same region and the same baseline volume tertile (0-430, 431-811, and ≥812 admissions for MJRLE in 2013). Automated matching was restricted to PGPs with a log odds propensity score absolute difference below 0.5. We then hand-matched 20 practices (14 large, 4 medium, and 2 small) by removing the within-region match requirement and selecting the remaining potential control within the same volume group with the closest number of surgeons in the practice (the dominant factor in the propensity model). Any practice or market characteristic with a standardized mean difference (SMD) of 0.2 or higher after matching was included in the regression models described below as a covariate.

### Patient Identification

We used 100% Medicare inpatient files from January 1, 2013, to September 30, 2017, to identify index admissions for MJRLE for which either the discharging clinician or the operating clinician belonged to a participating PGP or to a control PGP, per CMS’s BPCI inclusion rules. We included only beneficiaries who were continuously enrolled in Parts A and B during their episode of care and excluded those with end-stage kidney disease.

We considered baseline to be January 1, 2013, through September 30, 2013, for all PGPs and controls. The intervention period varied, because PGPs could join BPCI on a rolling basis, and was set for each BPCI PGP and its matched controls as the time from 3 months postenrollment through the end of September 2017. For the 91 BPCI practices analyzed here, the mean intervention period was 22.9 months, or just over 7 quarters.

### Outcomes

Our primary outcome was the change in Medicare Part A payments per episode. Because few PGPs chose 30-day or 60-day episodes, we analyzed 90-day episodes for all participants, as has been done previously.^1^ For each admission for MJRLE, standardized Medicare-allowed episode payments (which remove differences based on wage index and special payments, such as graduate medical education) for the initial hospital stay plus 90 days following discharge were calculated using 100% inpatient, skilled nursing facility (SNF), home health agency, and durable medical equipment claims. We refer to these as Medicare Part A payments and consider this our primary outcome. For the roughly 20% of patients for whom we had Part B outpatient and physician claims (based on a 20% random sample nationally), we also calculated 90-day standardized Part B payments. Total payments were Winsorized at the 95th percentile of national episode payments annually per CMS specifications and adjusted for inflation to prices in 2017.

Secondary outcomes included changes in patient selection, measured by the mean Medicare Chronic Conditions Warehouse comorbidity score; per-PGP quarterly volume; changes in key individual components of payment and Part B payments; and changes in 30-day and 90-day readmissions, 30-day and 90-day mortality, healthy days at home, and the proportion of patients discharged home, having any SNF stays, and using any home health services.

### Statistical Analysis

Market and PGP characteristics were compared between BPCI PGPs and their matched controls using SMDs. Our primary approach was an “intention to treat” approach, in which we included all participating PGPs regardless of whether they ultimately dropped out of the program, which they could do without penalty at any time. Our models are described in greater detail in the eMethods in the Supplement. We used a difference-in-differences (DID) approach to examine changes in each outcome in the baseline vs intervention period. To validate this approach, we evaluated preintervention slopes between BPCI PGPs and controls for Medicare Part A payments, readmissions, and mortality (Table 1). Analyses were run at the episode level, with each outcome in a separate model. Preintervention vs postintervention interacted with BPCI status was the primary predictor. A marginal, generalized equation approach to modeling was used to account for clustering within practices, along with fixed indicators for match groups. We controlled for diagnosis-related group, age, sex, and patient comorbidities using individual CMS Chronic Conditions Warehouse comorbidities. Practice size, volume, Medicare Advantage penetration, and the number of rehabilitation hospitals at the county level were also included owing to covariate imbalance after matching. We conducted 4 sensitivity analyses: First, we included only the practices that matched within our prespecified caliper and dropped all hand-matched groups. Second, we ran stratified models by volume tertile. Third, we conducted a treatment-on-the-treated analysis, dropping PGPs as they dropped from the program over time. Fourth, we used dummy variables for each quarter to control for secular trends.

**Table 1.** Baseline Practice and Market Characteristics

Characteristic	BPCI, mean	All non-BPCI, mean	SMD	Matched controls, mean	SMD
No. of practices	91	2951	NA	169	NA
Mean No. of surgeons^a^	26.9	3.8	3.037	14.2	0.655
Practice location, %					
Rural (non-CBSA)	0.02	0.04	0.020	0.01	0.037
Region^b^					
Northeast	17.6	19.7	0.054	25.4	0.188
Midwest	18.7	17.6	0.028	22.5	0.093
South	42.9	37.2	0.116	33.7	0.189
West	20.9	25.5	0.105	18.3	0.064
County characteristics^c^					
Population ≥65 y size	44 067	72 195	0.258	37 540	0.096
Median household income, $	53 923	53 680	0.019	54 853	0.078
% Medicare Advantage in county^a^	27.8	26.0	0.129	25.0	0.210
SNF beds per 10 000	1270	1871	0.235	1086	0.105
No. of rehabilitation hospitals^a^	0.26	0.24	0.040	0.12	0.326
PGP market share	0.07	0.03	0.432	0.07	0.003
Market concentration (HHI)	0.03	0.01	0.229	0.04	0.052
From claims					
Quarterly change of readmission rates, %	−0.1	−0.3	0.391	−0.2	0.213
Quarterly change of mortality rates, %	−0.0	−0.0	0.285	0.0	0.134
Quarterly change of payments/episode, $	−293	−231	0.279	−334	0.149
No. of baseline patient episodes per PGP^a^^,^^b^	771.5	107.3	2.931	504.2	0.472
Total No. of baseline patient episodes, all time periods^d^	210 999	NA	NA	250 599	NA
Age, No. (%)					
≤64 y	14 182 (6.7)	NA	NA	16 441 (6.6)	0.006
65-79 y	158 385 (75.1)	NA	NA	187 235 (74.7)	0.008
≥80 y	38 432 (18.2)	NA	NA	46 923 (18.7)	0.013
Female, No. (%)	133 022 (63.0)	NA	NA	158 192 (63.1)	0.002
Medicaid, No. (%)	15 244 (7.2)	NA	NA	17 857 (7.1)	0.004
Disabled without ESKD, No. (%)	29 116 (13.8)	NA	NA	34 538 (13.8)	0.000
Race/ethnicity, No. (%)					
White	191 675 (90.8)	NA	NA	227 944 (91.0)	0.004
Black	12 404 (5.9)	NA	NA	13 640 (5.4)	0.019
Hispanic	1006 (0.5)	NA	NA	1282 (0.5)	0.005
Unknown/other	5914 (2.8)	NA	NA	7733 (3.1)	0.017
CCW mean	3.82	NA	NA	3.96	0.056
Level of complexity, No. (%)					
DRG with MCC	4956 (2.3)	NA	NA	4786 (1.9)	0.030
DRG without CC	206 043 (97.7)	NA	NA	245 813 (98.1)	0.031
Patients with fracture, No. (%)	4425 (2.1)	NA	NA	4769 (1.9)	0.014

Abbreviations: BPCI, Bundled Payments for Care Improvement; CBSA, core-based statistical area (defined as metropolitan and micropolitan statistical areas); CC, complication or comorbidity; CCW, Chronic Conditions Warehouse, a Medicare-supplied comorbidity measure that ranges from 0 to 27, with higher scores indicating more comorbidities; DRG, diagnosis-related group; ESKD, end-stage kidney disease; HHI, Herfindahl-Hirschman Index (defined as the sum of the squares of each PGP’s market share, such that a perfectly competitive market has an HHI near 0, and a completely concentrated market has an HHI of 1); MCC, major complication or comorbidity; NA, not applicable; PGP, physician group practice; SMD, standardized mean difference; SNF, skilled nursing facility.

^a^ Included in regression models for payments, patient selection, and outcomes.

^b^ Included as a stratum for matching.

^c^ All county-level characteristics are from 2013, except the number of rehabilitation hospitals, which is from 2012. Markets are defined using counties.

^d^ Number of episodes includes the baseline period, the 3 months before (burn-in) or after (burn-out) each PGP’s date of enrollment in BPCI, and the intervention period. See eMethods in the Supplement for more details about the model. Presented characteristics represent individual patient episodes. Patients may have had multiple episodes, so long as they were more than 90 days apart.

For our primary outcome, the change in Medicare Part A payments per episode, a 2-tailed *P* value less than 0.05 was considered statistically significant. Secondary end points and subgroup analyses should be considered exploratory. Analyses were performed using SAS, version 9.4 (SAS Institute).

## Results

### PGP and Market Characteristics

There were 91 orthopedic PGPs that participated in BPCI Model 2 and 2951 that did not. Of these, the BPCI PGPs were successfully matched to 169 controls (Table 1). There were 74 343 patient episodes in the baseline period and 102 790 during the intervention in BPCI practices, and 88 147 patient episodes in the baseline period and 120 253 during the intervention in control practices; 291 214 of 461 598 patients (63.1%) were women, and 419 619 (90.9%) were White. The BPCI PGPs were larger and more often urban than nonparticipating PGPs. After matching, these differences were smaller, but BPCI PGPs still had a higher number of clinicians (mean surgeons per practice, 26.9 vs 14.2; SMD, 0.655) and patients (mean patients in the baseline period, 771.5 vs 504.2; SMD, 0.472) than controls. Baseline trends in readmission rates (−0.1% per quarter vs −0.2% per quarter; SMD, 0.213), mortality rates (0.0% per quarter vs 0.0% per quarter; SMD, 0.134), and total Medicare-allowed payments (−$293 per quarter vs −$334 per quarter; SMD, 0.149) were well matched, as were patient characteristics.

### Changes in Medicare Payments

Raw Medicare payments were well matched between BPCI and control PGPs in the baseline period but diverged as PGPs joined the BPCI program over the course of 2014 (Figure). At baseline, mean episode Medicare Part A payments among BPCI PGPs were $18 257, which decreased to $15 320 during the intervention, while control PGPs decreased from $17 927 to $16 170 (DID, −$1180; 95% CI, −$1565 to −$795; *P* < .001) (Table 2). There were no differential changes in mean episode Medicare Part B payments. Spending on the initial hospitalization and on readmissions was essentially unchanged in both groups, but spending on SNF stays decreased more in BPCI PGPs than controls (−$1820 vs −$910; DID, −$911; 95% CI, −$1207 to −$614). Home health spending and durable medical equipment spending also decreased more in BPCI PGPs compared with controls.

**Figure.**
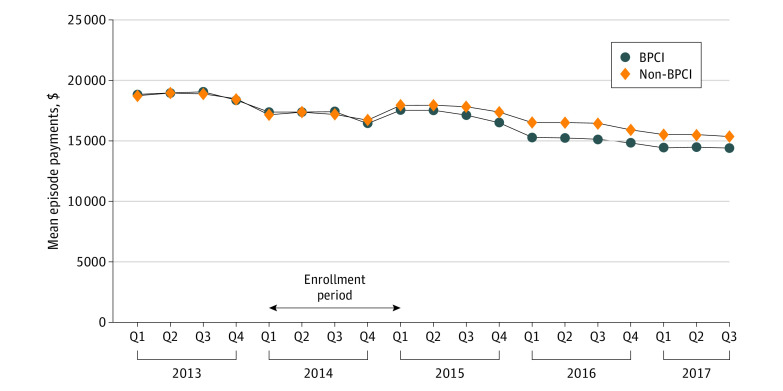
Raw Mean Episode Medicare-Allowed Payments for BPCI Participants vs Nonparticipants and Quarterly Program Enrollment BPCI indicates Bundled Payments for Care Improvement.

**Table 2.** Changes in Medicare Payments per Episode of Major Joint Replacement of the Lower Extremity

Cost^a^	$							*P* value
	BPCI			Matched controls			DID estimate (95% CI)	
	Baseline	Intervention	Diff	Baseline	Intervention	Diff		
No. of episodes^b^	74 343	102 790	NA	88 147	120 253	NA	NA	NA
Medicare Part A payments	18 257	15 320	−2937	17 927	16 170	−1757	−1180 (−1565 to −795)	<.001
Initial hospital stay	11 843	11 800	−44	11 660	11 479	−181	138 (−47 to 322)	.14
SNF stays	3466	1646	−1820	3155	2246	−910	−911 (−1207 to −614)	<.001
Readmission	902	793	−109	924	900	−24	−85 (−142 to −28)	.003
IRF	43	24	−19	46	31	−15	−4 (−25 to 18)	.72
Long-term care hospital	77	55	−22	97	104	7	−30 (−48 to −11)	.002
Home health agency	1851	960	−891	1971	1356	−615	−276 (−422 to −131)	<.001
DME	76	43	−33	74	54	−21	−12 (−22 to −3)	.01
Part B payments (physician and outpatient)^c^	2384	2489	105	2490	2616	126	−21 (−95 to 54)	.54

Abbreviations: BPCI, Bundled Payments for Care Improvement; DID, difference in differences; diff, difference; DME, durable medical equipment; IRF, inpatient rehabilitation facility; NA, not applicable; SNF, skilled nursing facility.

^a^ Costs are adjusted using patient-level comorbidities from Medicare’s Chronic Conditions Warehouse (a Medicare-supplied comorbidity measure that ranges from 0 to 27, with higher scores indicating more comorbidities) data.

^b^ Number of episodes only counts patients in the baseline or intervention time period and does not include episodes 3 months before (burn-in) or after (burn-out) each physician group practice’s date of enrollment in BPCI. See eMethods in the Supplement for more details about the model.

^c^ Calculated from the 20% file rather than the 100% file; not included in total Medicare Part A payments.

These findings were similar in both the fracture and nonfracture subgroups. Overall spending was much higher in the MJRLE with fracture group, but statistically significant reductions in spending were seen for each (for fracture, a reduction of $3400 vs $1088; DID, −$2313; 95% CI, −$3564 to −$1061; and for nonfracture, a reduction of $2849 vs $1679; DID, −$1170; 95% CI, −$1557 to −$786; interaction *P* = .18) (eTable 1 in the Supplement). In sensitivity analyses, similar patterns were seen when limiting the sample to automatically matched controls (eTable 2 in the Supplement), when stratifying by volume (eTable 3 in the Supplement), when estimating treatment-on-the-treated effects (eTable 4 in the Supplement), and when including time dummies (eTable 5 in the Supplement).

### Changes in Volume, Case Mix, and Clinical Outcomes

Volume per quarter for MJRLE was unchanged for both BPCI participants and controls over time (Table 3), as were all measures of patient selection with the exception of the proportion of patients younger than 65 years and who qualified for Medicare on the basis of a disability, which decreased to a greater degree in BPCI PGPs than controls. Findings were similar in the fracture and nonfracture subgroups (eTable 6 in the Supplement).

**Table 3.** Changes in Volume and Case Mix

Variable	No. (%)						
	BPCI			Matched controls			DID estimate (95% CI)
	Baseline	Intervention	Diff	Baseline	Intervention	Diff	
No. of episodes^a^	74 343	102 790	NA	88 147	120 253	NA	NA
No. of cases per quarter	120.5	138.3	17.9	77.7	89.6	11.9	6.0 (−2.1 to 14.1)
Age							
≤64 y	5518 (7.4)	6354 (6.2)	−1.2	6072 (6.9)	7539 (6.3)	−0.6	−0.62 (−1.06 to −0.18)
65-79 y	54 518 (73.3)	78 450 (76.3)	3.0	64 740 (73.4)	91 041 (75.7)	2.3	0.73 (−0.15 to 1.60)
≥80 y	14 307 (19.2)	17 986 (17.5)	−1.7	17 335 (19.7)	21 673 (18.0)	−1.6	−0.10 (−0.93 to 0.72)
Female	47 472 (63.9)	64 365 (62.6)	−1.2	56 282 (63.9)	75 449 (62.7)	−1.1	−0.13 (−0.84 to 0.58)
Medicaid	5860 (7.9)	6957 (6.8)	−1.1	6667 (7.6)	8230 (6.8)	−0.7	−0.39 (−0.92 to 0.13)
Disabled without ESKD	10 870 (14.6)	13 563 (13.2)	−1.4	12 406 (14.1)	16 191 (13.5)	−0.6	−0.82 (−1.44 to −0.20)
Race/ethnicity^b^							
White	67 832 (91.2)	93 021 (90.5)	−0.7	80 746 (91.6)	108 800 (90.5)	−1.1	0.38 (−0.13 to 0.90)
Black	4528 (6.1)	5930 (5.8)	−0.3	4701 (5.3)	6595 (5.5)	0.2	−0.47 (−0.97 to 0.02)
Hispanic	346 (0.5)	486 (0.5)	0.0	470 (0.5)	609 (0.5)	0.0	0.03 (−0.08 to 0.15)
Unknown/other	1637 (2.2)	3353 (3.3)	1.1	2230 (2.5)	4249 (3.5)	1.0	0.06 (−0.29 to 0.40)
CCW mean	3.84	3.80	−0.04	3.98	3.96	−0.02	−0.03 (−0.08 to 0.02)
Level of complexity							
DRG with MCC	1968 (2.6)	2205 (2.1)	−0.5	1893 (2.1)	2068 (1.7)	−0.4	−0.1 (−0.4 to 0.3)
DRG without CC	72 375 (97.4)	100 585 (97.9)	0.5	86 254 (97.9)	118 185 (98.3)	0.4	0.1 (−0.3 to 0.4)
Patients with fracture	2021 (2.7)	1684 (1.6)	−1.1	2228 (2.5)	1573 (1.3)	−1.2	0.1 (−0.3 to 0.6)

Abbreviations: BPCI, Bundled Payments for Care Improvement; CCW, Chronic Conditions Warehouse, a Medicare-supplied comorbidity measure that ranges from 0 to 27, with higher scores indicating more comorbidities; DID, difference in differences; diff, difference; DRG, diagnosis-related group (DRG without CC is a given diagnosis without complication or comorbidity; DRG with MCC is a given diagnosis with major complication or comorbidity); ESKD, end-stage kidney disease; NA, not applicable.

^a^ Number of episodes only counts patients in the baseline or intervention time period and does not include episodes 3 months before (burn-in) or after (burn-out) each physician group practice’s date of enrollment in BPCI. See eMethods in the Supplement for more details about the model.

^b^ Race/ethnicity is defined using Medicare enrollment data.

There were no differential changes in 30-day or 90-day mortality rates or emergency department visits without hospitalization between BPCI PGPs and controls. There was a greater decrease in 30-day and 90-day readmission rates among BPCI PGPs (90-day, BPCI, 8.7% to 7.5%; vs controls, 8.9% to 8.7%; DID, −1.0%; 95% CI, −1.4% to −0.5%). The BPCI PGPs also had a relative increase in healthy days at home (BPCI, 82.9 to 84.8; vs controls, 83.1 to 84.4; DID, 0.6; 95% CI, 0.4 to 0.8) (Table 4). There were also significant differences in utilization, with BPCI PGPs having large differential increases in the proportion of patients discharged home (23.6% to 43.4% vs 22.2% to 31.8%; DID, 10.2% [6.2% to 14.1%]), and decreases in the proportion using any SNF or home health agency services.

**Table 4.** Changes in Clinical Outcomes and Utilization

Outcome^a^	Risk-adjusted rate						
	BPCI			Matched controls			DID estimate (95% CI)
	Baseline	Intervention	Diff	Baseline	Intervention	Diff	
No. of episodes included^b^	74 343	102 790	NA	88 147	120 253	NA	NA
Readmission							
30 d	4.2	3.8	−0.4	4.3	4.2	−0.1	−0.3 (−0.6 to 0.0)
90 d	8.7	7.5	−1.2	8.9	8.7	−0.2	−1.0 (−1.4 to −0.5)
Mortality							
30 d	0.3	0.2	−0.1	0.2	0.2	0.0	−0.1 (0.0 to 0.0)
90 d	0.5	0.5	−0.1	0.4	0.5	−0.1	−0.2 (0.0 to 0.0)
ED visits without hospitalization							
30 d	7.1	7.7	0.7	6.9	7.5	0.5	0.1 (−0.7 to 0.9)
90 d	12.0	12.6	0.5	12.4	12.9	0.4	0.1 (−0.8 to 1.0)
Healthy days at home, risk-adjusted No.	82.9	84.8	1.9	83.1	84.4	1.3	0.6 (0.4 to 0.8)
Discharged home	23.6	43.4	19.9	22.2	31.8	9.7	10.2 (6.2 to 14.1)
With any SNF stay	33.9	20.7	−13.2	30.5	23.0	−7.5	−5.7 (−8.0 to −3.5)
With any HHA usage	58.6	47.2	−11.4	62.5	60.0	−2.5	−8.9 (−13.3 to −4.5)

Abbreviations: BPCI, Bundled Payments for Care Improvement; DID, difference in differences; diff, difference; ED, emergency department; HHA, home health agency; NA, not applicable; SNF, skilled nursing facility.

^a^ Outcomes are adjusted using patient-level comorbidities from Medicare’s Chronic Conditions Warehouse (a Medicare-supplied comorbidity measure that ranges from 0 to 27, with higher scores indicating more comorbidities) data.

^b^ Number of episodes only counts patients in the baseline or intervention time period and does not include episodes 3 months before (burn-in) or after (burn-out) each physician group practice’s date of enrollment in BPCI. See eMethods in the Supplement for more details about the model.

In exploratory subgroup analyses, among patients who had MJRLE with fracture, there were relative decreases at BPCI PGPs vs controls in 30-day mortality (4.5% to 2.7% vs 4.4% to 5.0%; DID, −2.3%; 95% CI, −4.1% to −0.6%) (eTable 7 in the Supplement). Among patients without fracture undergoing MJRLE, there was no change in 30-day or 90-day mortality or emergency department visits, but significant reductions in 30-day and 90-day readmission rates (90-day, 8.4% to 7.3%; vs 8.7% to 8.5%; DID, −1.0%; 95% CI, −1.4% to −0.5%) and postacute care utilization and increases in healthy days at home.

## Discussion

We found that PGP participation in BPCI for MJRLE was associated with significant reductions in overall Medicare payments, with no deleterious changes in clinical outcomes. Savings were largely driven by reductions in SNF payments, both in patients with and without fractures undergoing MJRLE .

The intent of BPCI was to incentivize clinicians to reduce unnecessary services and prevent avoidable readmissions. Results of the current study suggest that orthopedic surgery practices that joined BPCI were able to reduce SNF use following MJRLE without increasing readmissions. While SNF spending dropped in both subgroups, the proportion of patients discharged to SNF did not decline in the subset of patients undergoing MJRLE for fracture, who were older and sicker than their elective surgery counterparts. This suggests that reductions in SNF use were concentrated among the healthiest patients, where such care may not have been necessary. However, long-term functional status data are needed to determine whether reductions in SNF use might be associated with worse long-term outcomes in mobility or pain.

We found a small relative decrease in the proportion of patients undergoing surgery with BPCI PGPs who qualified for Medicare on the basis of a disability. There were no other changes that might suggest broad risk avoidance, including in comorbidity scores, the proportion of patients of minority race/ethnicity, or the proportion dually enrolled in Medicaid. Claims data cannot discern the appropriateness of any of the procedures examined, but we did not see changes in volume that suggest patient shifting as the major source of savings under the program.

The findings we document among PGPs participating in BPCI are similar or larger than those documented among hospitals and SNFs participating in the program,^1,3^ although direct comparisons are not feasible owing to the many differences between the patients seen in each setting. For example, the patients enrolled in BPCI via PGPs were markedly less likely to have a fracture and were younger and less sick than prior reports of BPCI patients in hospitals or at postacute care facilities.^3,10^ Physician group practices may have advantages in achieving savings, including long-term relationships with patients that might lead to greater comfort sending patients home rather than to SNF care, or to better-aligned financial incentives, because few PGPs have ownership relationships with postacute facilities, unlike hospitals. We did not see an increase in outpatient spending to suggest that PGPs added clinic visits to achieve reductions in SNF care, although it is possible that nonbilled services, such as phone calls, home visits, or remote monitoring, may have been provided.

To our knowledge, there have been no prior studies of PGP performance in BPCI published in the peer-reviewed literature. There have been 3 annual federal evaluations of BPCI; despite differences in methodology from the approach we used, they also found lower costs for patients cared for under BPCI.^10,11,12^ Additionally, there have been national and single-center studies showing savings for MJRLE among hospitals participating in BPCI Model 2.^1,13,14,15^ This study lends further support to the notion that episode-based payments for joint replacement can save money without adversely affecting patients.

### Limitations

There are limitations to the study findings. The BPCI is a voluntary program; generalizability to mandatory models is uncertain. We focused on orthopedic PGPs joining the MJRLE bundle because it was the most commonly selected, with adequate volume for analysis, and because identifying control practices is easier when examining a single specialty. These findings may not generalize to other types of practices or bundles. Our lists of participants were obtained from Medicare and not verified by PGPs, which could introduce error. While we included NPIs assigned to each PGP based on annual CMS data files, it is possible that individual clinicians changed practice affiliation midyear, which would lead to misclassification. As Medicare has not released data on target pricing nor on PGPs’ savings or losses under the program, we could only evaluate BPCI’s impact on patients and their outcomes, and not its overall financial impact for PGPs or to the federal government. The Center for Medicare & Medicaid Innovation publication^4^ noting a lack of net savings under BPCI did not break their findings out by hospital vs physician group or postacute care facility participation. We used a short preintervention baseline period; during this period, PGPs were surely aware they were nearing the beginning of the intervention and may have been preparing to redesign care. However, the finding that pretrends were similar between intervention and control PGPs is reassuring. We had a limited follow-up period, and longer-term follow-up may be necessary to evaluate how care evolves.^16^ Finally, we do not know how PGPs achieved the changes that we observed; this is an important area for future mixed-methods and qualitative research.

## Conclusions

In this cross-sectional study of US Medicare data, PGP participation in BPCI for MJRLE was associated with reductions in Medicare payments and improvements in clinical outcomes. It is unclear if these results would generalize outside this voluntary model.

## Notes

Supplement.
eMethods.
**eTable 1.** Change in Medicare Payments by fracture/no fracture subgroup **eTable 2.** Change in Medicare Payments compared to automatically matched controls only **eTable 3.** Change in Medicare Payments by volume strata **eTable 4.** Change in Medicare Payments, treatment on the treated approach **eTable 5.** Change in Medicare Payments using time dummies **eTable 6.** Changes in Volume and Case Mix by fracture/no fracture subgroup **eTable 7.** Changes in Clinical Outcomes by fracture/no fracture subgroup
