# Supplementary material for: Association of Physician Group Practice Participation in Bundled Payments With Patient Selection, Costs, and Outcomes for Joint Replacement
Source: JAMA Health Forum. 2021 May 6;2(5):e210295. doi: 10.1001/jamahealthforum.2021.0295 (PMC8796976; doi:10.1001/jamahealthforum.2021.0295)
Supplement: Supplement. — eMethods. eTable 1. Change in Medicare Payments by fracture/no fracture subgroup eTable 2. Change in Medicare Payments compared to automatically matched controls only eTable 3. Change in Medicare Payments by volume strata eTable 4. Change in Medicare Payments, treatment on the treated approach eTable 5. Change in Medicare Payments using time dummies eTable 6. Changes in Volume and Case Mix by fracture/no fracture subgroup eTable 7. Changes in Clinical Outcomes by fracture/no fracture subgroup [file jamahealthforum-e210295-s001.pdf]

Supplemental Online Content

Joynt Maddox KE, Orav EJ, Zheng J, Epstein AM. Association of physician group practice participation in bundled payments with patient selection, costs, and outcomes for joint replacement. *JAMA Health Forum.* 2021;2(5):e210295.
doi:10.1001/jamahealthforum.2021.0295

eMethods.

eTable 1. Change in Medicare Payments by fracture/no fracture subgroup

eTable 2. Change in Medicare Payments compared to automatically matched controls only

eTable 3. Change in Medicare Payments by volume strata

eTable 4. Change in Medicare Payments, treatment on the treated approach

eTable 5. Change in Medicare Payments using time dummies

eTable 6. Changes in Volume and Case Mix by fracture/no fracture subgroup

eTable 7. Changes in Clinical Outcomes by fracture/no fracture subgroup

This supplemental material has been provided by the authors to give readers additional information about their work.

© 2021 Joynt Maddox et al. *JAMA Health Forum.*

eMethods

A. Identifying BPCI practices

We obtained publicly available data from CMMI listing BPCI participants along with their start dates for financial incentives and the date they terminated participation. There were 154 practices that signed up for MJRLE. The participation data includes practice name and location, but not tax identification numbers (TINs). We used practice name and location to find each practice in the Medicare Data on Provider Practice and Specialty (MD-PPAS) dataset, which we were able to do for 150 of these 154 practices.

The remaining 4 practices were manually searched online, and physician lists were obtained from their websites. NPI numbers were then identified from public data sources (for example, NPI number lookup https://www.npinumberlookup.org/) for these physicians, and the MD-PPAS dataset was queried to find practices that contained those NPIs. For example, there was a group listed in the CMS BPCI enrollment data called "Maple Orthopedics" in Austin, TX (not its real name/location, just provided for the purpose of this illustration). This practice did not match by name and address to the MD-PPAS data. We searched for their practice online, and identified their website. From their practice website, we identified 10 surgeons, and searched for their NPIs using a public NPI lookup tool. We then searched for those 10 NPIs in MD-PPAS. All 10 matched to a legal business name called "Orthopedic Associates of Austin" also located in Austin, TX at the address associated with "Maple Orthopedics" in the CMS data. In another situation, a practice name was listed in the CMS data as "The Orthopaedic Group, LLC". Searching the practice's name and address online yielded a website for "The Orthopedic Group, LLC" at the same address, which was then located in MD-PPAS with that alternate spelling.

However, when we linked these 154 TINs back to the claims data, we discovered that many of the PGPs that signed up for the MJRLE group in BPCI were large multispecialty or hospitalist practices that signed up for many conditions (e.g. heart failure, pneumonia), and did not actually care for MJRLE patients under the same TIN – only 96 of these practices ever billed for even a single MJRLE on an inpatient basis as the operating or attending surgeon over the study period, although they had many other attributed hospitalizations. We were therefore concerned that these multispecialty practices might have complex billing arrangements under multiple TINs that were impacting our ability to correctly identify them. However, of the 96 practices with one or more MRJLE episodes, 93 were orthopedic surgery practices. We therefore limited the analytic sample, both for BPCI participants and potential controls, to orthopedic surgery practices to enable more appropriate comparisons and because we were confident that the data reflected actual participation in the BPCI model.

We did additional manual verification of the orthopedic practices, in part because many were quite large and we wanted to be sure that the data were accurate. For example, our largest participating practice, located in the Northwest, had 360 NPIs affiliated with its identified TIN per year on average in the MD-PPAS data during our study period, of which 199 were surgeons. We confirmed that as of early 2021, this group practice has over a hundred clinic locations and over 400 clinicians in its online directory, including over 200 surgeons. Our second largest participating practice, located in the Southeast, had 346 NPIs affiliated with its identified TIN per year on average in the MD-PPAS data during our study period, of which 146 were surgeons. Its online directory included over 30 locations and over 400 clinicians that would likely have unique NPIs, including over 140 physicians, as well as physician assistants, physical therapists, hand therapists, occupational therapists, and trainers. Other hand-checked practices across a range of practice sizes were similarly comparable between the MD-PPAS and online-searched data.

© 2021 Joynt Maddox et al. *JAMA Health Forum.*

Of the 93 orthopedic surgery practices that thus comprised our sample, 91 were successfully matched to control practices and comprised our analytic sample. Start dates for these practices were as follows: 1/1/2014: 2 practices; 1/1/2015: 21 practices; 4/1/2015: 18 practices; 7/1/2015: 48 practices; 10/1/2015: 2 practices.

B. Propensity Matching

Using propensity scores based on PGP and market characteristics, each BPCI PGP was matched without replacement with up to 3 control orthopedic PGPs within the same region and the same baseline volume tertile (0-430, 431-811, and 812 or more admissions for MJRLE in 2013). Automated matching was restricted to PGPs with a log odds propensity score absolute difference below 0.5. We then hand-matched 20 practices (14 large, 4 medium, and 2 small) by removing the within-region match requirement and selecting the remaining potential control within the same volume group with the closest number of surgeons in the practice (the dominant factor in the propensity model). Any practice or market characteristic with an SMD of 0.2 or higher after matching was included in the regression models described below as a covariate.

C. Analyses

The regression model described in the methods section was implemented using a marginal, generalized equation approach (the GENMOD procedure in the SAS statistical package). The GEE approach is robust in that a particular distribution for the outcome variable is not required to be specified, only that the distribution be within the exponential family, which includes many common distributions including normal, gamma, and logistic. And the approach does not require specification of the correlation structure since the correlation is estimated empirically from the model residuals. We did however specify an independent working correlation structure so that each patient would count equally in the effect estimates and so that imbalances in samples sizes between practices would not create a bias. Regardless of the specified working correlation, the effect estimates will be consistent as long as there are a sufficient number of practices. We also assumed a linear model with an identity link for the mean function, so that covariates would have additive rather than multiplicative effects on the outcome. Absolute rather than relative changes in costs, as well as in outcome rates, are simpler to interpret and are the conventional way to present the results of policy interventions. To reduce the impact of outliers, costs were Winsorized, in concordance with CMS conventions.

In addition to adjusting for correlation within practices, the model included fixed effects for match groups so that the effect of the intervention was estimated solely by comparing each BPCI practice to their matched controls. The primary predictors in the model were an indicator for time period (pre- versus post-intervention), intervention group (BPCI versus matched control) and the interaction between these two indicators. The interaction term determined whether the change in outcome was greater for BPCI practices than for their matched controls. The model also adjusted for temporal trends by including a linear term for calendar quarter, and for differences between practices by including fixed effects for practice characteristics which were not balanced by the matching algorithm: number of patients, number of surgeons, percentage Medicare Advantage penetration, and the number of rehabilitation hospitals at the county level. Differences in patients seen at different practices were

© 2021 Joynt Maddox et al. *JAMA Health Forum.*

accounted for by fixed effects for DRG, patient age, patient gender, and the 27 Chronic Condition Warehouse (CCW) co-morbidities.

The primary model equation is:

> Expected Total payments = Intercept + TimePeriod + BPCI + TimePeriod*BPCI + Calendar-Quarter + Match-Group(1-91) + Number-of-Surgeons + Number of Patients + Medicare-Advantage-Penetration + Rehabilitation-Hospitals + Patient-Age + Patient-Gender + DRG (1-2) + CCW (1-27)

Where TimePeriod includes four group-specific time frames: pre-intervention, burn-in, burn-out, and post-intervention. For each PGP, patients with procedures between 1/1/2013 and 9/30/2013 constitute the reference pre-intervention period and patients seen from 10/1/2013 until the participation date of the BPCI-A PGP constitute the burn-in period. Patients from the participation date until three months later constitute the burn-out period, and those seen from three months post-joining until 9/30/2017 constitute the post-intervention period. Therefore, the post-intervention period is specific to each match group, as are burn-in and burn-out periods surrounding the participation date.

In a sensitivity analysis, calendar quarter was added to the model as a categorical predictor to account for changes over time. Because of the redundancy between this time variable and the study TimePeriods, changes between TimePeriods could not be estimated in the control practices. However, the interaction between TimePeriod and BPCI (i.e., the diff-in-diffs effect) could still be estimated and tested.

D. Model coefficients

Model coefficients for the primary outcome (Medicare payments per episode) are shown below.

© 2021 Joynt Maddox et al. *JAMA Health Forum.*

Full model output:

Parameter			Estimate	Standard Error	95% Confidence Limits		Z	Pr > |Z|
**Intercept**			660.03	1124.94	-1544.82	2864.88	0.59	0.557
**timeperiod3**	Burnin		-265.25	119.76	-499.98	-30.52	-2.21	0.027
**timeperiod3**	Burnout		-511.85	93.14	-694.40	-329.31	-5.50	<.0001
**timeperiod3**	Intervention		-1757.43	121.45	-1995.46	-1519.40	-14.47	<.0001
**timeperiod3**	Baseline		0.00	0.00	0.00	0.00	.	.
**bpciTIN1**	1		330.09	222.22	-105.45	765.63	1.49	0.137
**bpciTIN1**	0		0.00	0.00	0.00	0.00	.	.
**timeperiod3*bpciTIN1**	Burnin	1	-759.65	179.37	-1111.21	-408.10	-4.24	<.0001
**timeperiod3*bpciTIN1**	Burnin	0	0.00	0.00	0.00	0.00	.	.
**timeperiod3*bpciTIN1**	Burnout	1	-282.12	154.38	-584.69	20.45	-1.83	0.068
**timeperiod3*bpciTIN1**	Burnout	0	0.00	0.00	0.00	0.00	.	.
**timeperiod3*bpciTIN1**	Intervention	1	-1180.04	196.23	-1564.64	-795.44	-6.01	<.0001
**timeperiod3*bpciTIN1**	Intervention	0	0.00	0.00	0.00	0.00	.	.
**timeperiod3*bpciTIN1**	Baseline	1	0.00	0.00	0.00	0.00	.	.
**timeperiod3*bpciTIN1**	Baseline	0	0.00	0.00	0.00	0.00	.	.
**Age**			208.54	4.53	199.65	217.43	45.99	<.0001
**Sex**	1		-1291.82	44.68	-1379.38	-1204.26	-28.92	<.0001
**Sex**	2		0.00	0.00	0.00	0.00	.	.
**Black**			1510.07	113.32	1287.97	1732.18	13.33	<.0001
**Hispanic**			1412.40	245.45	931.33	1893.48	5.75	<.0001
**Other**			155.55	103.59	-47.49	358.59	1.50	0.133
**Acute MI**			376.95	270.22	-152.66	906.57	1.40	0.163
**Alzheimer's**			1187.89	230.18	736.75	1639.03	5.16	<.0001
**Dementia**			4234.59	151.62	3937.41	4531.77	27.93	<.0001
**Atrial fibrillation**			832.08	68.43	697.97	966.19	12.16	<.0001
**Cataract**			-418.00	28.04	-472.96	-363.03	-14.91	<.0001
**Chronic kidney disease**			915.23	45.77	825.51	1004.94	19.99	<.0001

© 2021 Joynt Maddox et al. *JAMA Health Forum.*

**COPD**			1438.98	69.56	1302.64	1575.31	20.69	<.0001
**Heart failure**			1865.93	70.28	1728.18	2003.68	26.55	<.0001
**Diabetes**			962.61	40.52	883.19	1042.02	23.76	<.0001
**Glaucoma**			-109.33	44.93	-197.40	-21.26	-2.43	0.015
**Hip fracture**			3010.60	200.02	2618.57	3402.64	15.05	<.0001
**Ischemic heart disease**			355.37	44.83	267.51	443.24	7.93	<.0001
**Depression**			1690.32	42.67	1606.69	1773.95	39.62	<.0001
**Osteoporosis**			842.26	59.96	724.74	959.78	14.05	<.0001
**Arthritis**			-149.82	42.44	-233.00	-66.63	-3.53	0.000
**Stroke or TIA**			1192.50	102.52	991.56	1393.44	11.63	<.0001
**Breast cancer**			-296.39	65.03	-423.84	-168.95	-4.56	<.0001
**Colorectal cancer**			33.03	125.62	-213.18	279.24	0.26	0.793
**Prostate cancer**			-322.14	71.49	-462.26	-182.02	-4.51	<.0001
**Lung cancer**			413.90	224.22	-25.57	853.37	1.85	0.065
**Endometrial cancer**			376.15	211.44	-38.27	790.58	1.78	0.075
**Anemia**			768.43	39.48	691.05	845.81	19.46	<.0001
**Asthma**			288.89	49.20	192.46	385.32	5.87	<.0001
**Hyperlipidemia**			-646.26	33.48	-711.88	-580.64	-19.30	<.0001
**Prostatic hypertrophy**			-110.12	53.72	-215.42	-4.82	-2.05	0.040
**Hypertension**			409.85	30.04	350.98	468.73	13.64	<.0001
**Hypothyroidism**			170.23	37.59	96.56	243.90	4.53	<.0001
**Number of admissions 2013**			-0.32	0.71	-1.71	1.07	-0.45	0.655
**Sum of n of surgeons**			1.49	2.76	-3.91	6.90	0.54	0.588
**Medicare advantage %**			-3.82	10.24	-23.89	16.25	-0.37	0.709
**N of rehab hospitals**			-629.08	263.91	-1146.33	-111.83	-2.38	0.017
**DRG code**	469		1674.87	184.64	1312.98	2036.77	9.07	<.0001
**DRG code**	470		0.00	0.00	0.00	0.00	.	.

Model also includes individual match group fixed effects that are not shown in the table.

© 2021 Joynt Maddox et al. *JAMA Health Forum.*

eTable 1. Change in Medicare Payments by fracture/no fracture subgroup

	BPCI			Matched Controls			Diff-in-Diff Estimate		
	Baseline	Intervention	Diff	Baseline	Intervention	Diff	DID	Lower 95% CI	Upper 95% CI
**MJRLE with Fracture**									
**N of episodes**	2021	1684		2228	1573				
**Total payments**	$29,620	$26,219	-$3,400	$29,093	$28,006	-$1,088	-$2,313	-$3,564	-$1,061
**Index hosp**	$12,510	$12,112	-$398	$12,493	$12,267	-$226	-$172	-$337	-$7
**SNF stays**	$12,744	$10,216	-$2,528	$12,208	$11,291	-$917	-$1,611	-$2,516	-$706
**Readmission**	$2,464	$2,368	-$97	$2,161	$2,321	$160	-$257	-$774	$260
**IRF**	$122	-$36	-$158	$191	$250	$59	-$217	-$494	$59
**LTCH**	$215	$250	$35	$352	$378	$26	$9	-$247	$264
**HHA**	$1,523	$1,281	-$242	$1,642	$1,470	-$172	-$70	-$220	$80
**DME**	$41	$29	-$12	$46	$29	-$17	$5	$0	$11
**Part B outpatient***	$2,906	$2,942	$36	$2,912	$3,160	$248	-$213	-$796	$371
**MJRLE without Fracture**									
**N of episodes**	72322	101106		85919	118680				
**Total payments**	$17,971	$15,122	-$2,849	$17,665	$15,986	-$1,679	-$1,170	-$1,557	-$786
**Index hosp**	$11,826	$11,793	-$33	$11,640	$11,467	-$173	$140	-$47	$327
**SNF stays**	$3,230	$1,490	-$1,741	$2,945	$2,106	-$839	-$902	-$1,195	-$609
**Readmission**	$863	$766	-$97	$893	$877	-$16	-$81	-$139	-$24
**IRF**	$40	$24	-$16	$43	$29	-$15	-$1	-$22	$19
**LTCH**	$75	$52	-$22	$90	$99	$10	-$32	-$50	-$14
**HHA**	$1,861	$955	-$906	$1,979	$1,354	-$625	-$281	-$429	-$133
**DME**	$76	$43	-$33	$75	$54	-$21	-$12	-$22	-$3
**Part B outpatient***	$2,372	$2,481	$109	$2,480	$2,607	$127	-$18	-$93	$58

BPCI=Bundled Payments for Care Improvement; DID=difference in differences; DME=durable medical equipment; HHA=home health agency; IRF=inpatient rehabilitation facility; LTCH=long-term care hospital; MJRLE=major joint replacement of the lower extremity; SNF=skilled nursing facility. Costs are adjusted using patient-level comorbidities from Medicare's chronic conditions warehouse (CCW) data. * Calculated from 20% rather than 100% files, not included in total payments.

© 2021 Joynt Maddox et al. *JAMA Health Forum.*

eTable 2. Change in Medicare Payments compared to automatically matched controls only

	BPCI			Matched Controls			Diff-in-Diff Estimate		
	Baseline	Intervention	Diff	Baseline	Intervention	Diff	DID	Lower 95% CI	Upper 95% CI
MJRLE									
N of episodes	41988	56856		64632	86603				
Total payments	$18,549	$15,398	-$3,152	$17,976	$16,143	-$1,833	-$1,318	-$1,779	-$858
Index hosp	$11,846	$11,746	-$100	$11,810	$11,594	-$215	$116	-$123	$355
SNF stays	$3,687	$1,698	-$1,990	$3,105	$2,169	-$936	-$1,054	-$1,381	-$727
Readmission	$944	$792	-$152	$937	$908	-$29	-$123	-$192	-$53
IRF	$56	$27	-$29	$56	$35	-$21	-$8	-$39	$23
LTCH	$72	$51	-$21	$98	$105	$7	-$28	-$52	-$4
HHA	$1,873	$1,045	-$829	$1,892	$1,275	-$617	-$212	-$376	-$47
DME	$72	$39	-$33	$79	$57	-$22	-$11	-$20	-$2
Part B outpatient*	$2,342	$2,440	$98	$2,455	$2,583	$128	-$30	-$137	$78

BPCI=Bundled Payments for Care Improvement; DID=difference in differences; DME=durable medical equipment; HHA=home health agency; IRF=inpatient rehabilitation facility; LTCH=long-term care hospital; MJRLE=major joint replacement of the lower extremity; SNF=skilled nursing facility. Costs are adjusted using patient-level comorbidities from Medicare's chronic conditions warehouse (CCW) data. * Calculated from 20% rather than 100% files, not included in total payments.

© 2021 Joynt Maddox et al. *JAMA Health Forum.*

eTable 3. Change in Medicare Payments by volume strata

	BPCI			Matched Controls			Diff-in-Diff Estimate		
	Baseline	Intervention	Diff	Baseline	Intervention	Diff	DID	Lower 95% CI	Upper 95% CI
**Lowest tertile**									
**N of episodes**	6322	8888		15619	20863				
**Total payments**	$18,816	$16,012	-$2,804	$18,538	$16,405	-$2,133	-$671	-$1,413	$72
**Index hosp**	$11,770	$11,733	-$37	$11,802	$11,418	-$385	$348	-$26	$722
**SNF stays**	$3,395	$1,635	-$1,760	$3,480	$2,310	-$1,170	-$590	-$1,234	$54
**Readmission**	$917	$786	-$131	$938	$935	-$3	-$128	-$312	$57
**IRF**	$44	$9	-$35	$63	$41	-$22	-$13	-$69	$43
**LTCH**	$100	$91	-$9	$93	$119	$26	-$35	-$106	$36
**HHA**	$2,514	$1,712	-$802	$2,087	$1,530	-$558	-$245	-$503	$13
**DME**	$76	$46	-$30	$75	$54	-$21	-$9	-$26	$9
**Part B outpatient***	$2,292	$2,358	$65	$2,557	$2,649	$92	-$27	-$230	$177
**Middle tertile**									
**N of episodes**	19610	25311		34929	46639				
**Total payments**	$18,307	$14,970	-$3,337	$18,042	$16,293	-$1,749	-$1,588	-$2,204	-$971
**Index hosp**	$11,851	$11,739	-$112	$11,770	$11,626	-$144	$31	-$302	$365
**SNF stays**	$3,411	$1,433	-$1,979	$3,195	$2,258	-$936	-$1,042	-$1,453	-$631
**Readmission**	$907	$767	-$141	$913	$867	-$45	-$95	-$192	$2
**IRF**	$86	$38	-$48	$34	$30	-$4	-$44	-$86	-$2
**LTCH**	$90	$53	-$37	$99	$107	$8	-$45	-$82	-$9
**HHA**	$1,882	$900	-$982	$1,952	$1,351	-$601	-$380	-$592	-$168
**DME**	$80	$41	-$39	$79	$53	-$26	-$13	-$26	$1
**Part B outpatient***	$2,307	$2,472	$166	$2,408	$2,530	$122	$43	-$78	$164
**Highest tertile**									
**N of episodes**	48411	68591		37599	52751				
**Total payments**	$18,017	$15,216	-$2,800	$17,761	$16,136	-$1,626	-$1,175	-$1,752	-$597
**Index hosp**	$11,781	$11,760	-$21	$11,587	$11,455	-$133	$112	-$148	$372
**SNF stays**	$3,442	$1,675	-$1,767	$3,055	$2,275	-$780	-$987	-$1,474	-$500

© 2021 Joynt Maddox et al. *JAMA Health Forum.*

**Readmission**	$898	$805	-$93	$927	$915	-$13	-$80	-$158	-$2
**IRF**	$19	$14	-$5	$59	$37	-$22	$17	-$14	$49
**LTCH**	$67	$48	-$18	$100	$99	-$1	-$17	-$42	$8
**HHA**	$1,737	$872	-$865	$1,963	$1,302	-$661	-$205	-$427	$18
**DME**	$74	$42	-$31	$70	$54	-$16	-$15	-$29	-$1
**Part B outpatient***	$2,428	$2,516	$88	$2,540	$2,681	$142	-$54	-$158	$50

BPCI=Bundled Payments for Care Improvement; DID=difference in differences; DME=durable medical equipment; HHA=home health agency; IRF=inpatient rehabilitation facility; LTCH=long-term care hospital; MJRLE=major joint replacement of the lower extremity; SNF=skilled nursing facility. Costs are adjusted using patient-level comorbidities from Medicare's chronic conditions warehouse (CCW) data. * Calculated from 20% rather than 100% files, not included in total payments.

© 2021 Joynt Maddox et al. *JAMA Health Forum.*

eTable 4. Change in Medicare Payments, treatment on the treated approach

	BPCI			Matched Controls			Diff-in-Diff Estimate		
	**Baseline**	**Intervention**	**Diff**	**Baseline**	**Intervention**	**Diff**	**DID**	**Lower 95% CI**	**Upper 95% CI**
**MJRLE**									
**N of episodes**	72562	94495		88147	120253				
**Total payments**	$18,232	$15,388	-$2,844	$17,927	$16,168	-$1,759	-$1,085	-$1,502	-$668
**Index hosp**	$11,844	$11,834	-$10	$11,658	$11,478	-$181	$170	-$23	$364
**SNF stays**	$3,456	$1,686	-$1,769	$3,150	$2,240	-$910	-$859	-$1,174	-$544
**Readmission**	$917	$797	-$121	$920	$896	-$24	-$97	-$155	-$38
**IRF**	$39	$23	-$16	$47	$32	-$15	-$2	-$23	$20
**LTCH**	$79	$54	-$25	$97	$104	$7	-$32	-$51	-$14
**HHA**	$1,821	$953	-$868	$1,981	$1,365	-$616	-$252	-$395	-$109
**DME**	$75	$41	-$34	$74	$54	-$21	-$14	-$23	-$4
**Part B outpatient***	$2,398	$2,504	$105	$2,479	$2,606	$126	-$21	-$97	$55

BPCI=Bundled Payments for Care Improvement; DID=difference in differences; DME=durable medical equipment; HHA=home health agency; IRF=inpatient rehabilitation facility; LTCH=long-term care hospital; MJRLE=major joint replacement of the lower extremity; SNF=skilled nursing facility. Costs are adjusted using patient-level comorbidities from Medicare's chronic conditions warehouse (CCW) data. * Calculated from 20% rather than 100% files, not included in total payments.

© 2021 Joynt Maddox et al. *JAMA Health Forum.*

eTable 5. Change in Medicare Payments using time dummies

Condition:	Outcome	Difference in Differences (change in BPCI PGPs minus change in controls)	Lower CI	Upper CI
**MJRLE**	total payments	-$1,203	-$1,595	-$811
**MJRLE with Fracture**	total payments	-$2,292	-$3,530	-$1,055
**MJRLE without Fracture**	total payments	-$1,193	-$1,587	-$800

MJRLE=major joint replacement of the lower extremity. Costs are adjusted using patient-level comorbidities from Medicare's chronic conditions warehouse (CCW) data. Because time dummies are perfectly predictive of quarterly costs, mean costs in the baseline and intervention periods cannot be estimated from this model, and therefore only the difference in differences estimates are shown.

© 2021 Joynt Maddox et al. *JAMA Health Forum.*

eTable 6. Changes in Volume and Case Mix by fracture/no fracture subgroup

Variables	BPCI			Matched Controls			Diff-in-Diff Estimate		
	Baseline	Intervention	Diff	Baseline	Intervention	Diff	DID	Lower 95% CI	Upper 95% CI
**MJRLE with Fracture**									
**n cases per quarter**	5.14	4.05	-1.09	3.71	2.68	-1.03	-0.07	-0.83	0.70
**Age 64 and under**	3.5%	4.5%	0.9%	3.5%	5.2%	1.7%	-0.77%	-2.54%	0.99%
**Age 65-79**	33.5%	38.7%	5.2%	32.6%	37.8%	5.2%	-0.03%	-4.10%	4.04%
**Age 80+**	63.0%	56.9%	-6.2%	63.9%	57.0%	-7.0%	0.80%	-3.75%	5.36%
**Female**	72.8%	70.4%	-2.4%	73.1%	71.1%	-2.0%	-0.44%	-4.43%	3.55%
**Medicaid**	15.9%	13.8%	-2.1%	14.6%	15.8%	1.2%	-3.29%	-6.74%	0.17%
**Disabled w/o ESRD**	12.0%	12.7%	0.7%	10.7%	14.2%	3.5%	-2.82%	-5.69%	0.05%
**Race**									
**White**	96.5%	96.1%	-0.5%	96.5%	95.1%	-1.3%	0.89%	-0.72%	2.51%
**Black**	2.3%	2.9%	0.5%	2.5%	2.7%	0.2%	0.32%	-0.94%	1.58%
**Hispanic**	0.3%	0.2%	-0.1%	0.2%	0.2%	0.0%	-0.07%	-0.51%	0.37%
**Unknown/other**	0.8%	0.8%	0.0%	0.9%	2.0%	1.1%	-1.15%	-2.12%	-0.18%
**CCW mean**	4.69	4.81	0.12	4.70	4.80	0.10	0.02	-0.26	0.31
**Level of complexity**									
**DRG with MCC**	14.3%	16.8%	2.4%	13.2%	14.1%	0.9%	1.6%	-1.3%	4.4%
**DRG without CC**	85.7%	83.3%	-2.4%	86.8%	85.9%	-0.9%	-1.6%	-4.4%	1.3%
**MJRLE without Fracture**									
**n cases per quarter**	117.2	136.1	18.9	75.8	88.4	12.6	6.3	-1.8	14.4
**Age 64 and under**	7.5%	6.2%	-1.3%	7.0%	6.3%	-0.7%	-0.63%	-1.09%	-0.17%
**Age 65-79**	74.5%	77.0%	2.5%	74.5%	76.2%	1.7%	0.80%	-0.02%	1.62%
**Age 80+**	18.0%	16.8%	-1.2%	18.5%	17.5%	-1.0%	-0.17%	-0.94%	0.61%
**Female**	63.6%	62.5%	-1.1%	63.6%	62.6%	-1.0%	-0.14%	-0.86%	0.58%
**Medicaid**	7.7%	6.7%	-1.0%	7.4%	6.7%	-0.7%	-0.35%	-0.88%	0.18%
**Disabled w/o ESRD**	14.7%	13.2%	-1.5%	14.2%	13.5%	-0.7%	-0.78%	-1.42%	-0.15%
**Race**									
**White**	91.1%	90.4%	-0.7%	91.5%	90.4%	-1.1%	0.37%	-0.15%	0.90%
**Black**	6.2%	5.8%	-0.4%	5.4%	5.5%	0.1%	-0.49%	-1.00%	0.01%

© 2021 Joynt Maddox et al. *JAMA Health Forum.*

**Hispanic**	0.5%	0.5%	0.0%	0.5%	0.5%	0.0%	0.04%	-0.08%	0.16%
**Unknown/other**	2.2%	3.3%	1.1%	2.6%	3.6%	1.0%	0.08%	-0.27%	0.43%
**CCW mean**	3.82	3.78	-0.04	3.96	3.95	-0.01	-0.03	-0.08	0.02
**Level of complexity**									
**DRG with MCC**	2.3%	1.9%	-0.4%	1.9%	1.6%	-0.3%	-0.1%	-0.5%	0.2%
**DRG without CC**	97.7%	98.1%	0.4%	98.1%	98.4%	0.3%	0.1%	-0.2%	0.5%

BPCI=Bundled Payments for Care Improvement; CCW=chronic conditions warehouse, a Medicare-supplied comorbidity measure that ranges from 0-27 with higher scores indicating more comorbidities; DID=difference in differences; DRG=diagnosis-related group (DRG without CC is a given diagnosis without complication or comorbidity; DRG with CC is a given diagnosis with complication or comorbidity; and DRG with MCC is a given diagnosis with major complication or comorbidity); ESRD=end stage renal disease; MJRLE=major joint replacement of the lower extremity; SNF=skilled nursing facility. Race is defined using Medicare enrollment data.

© 2021 Joynt Maddox et al. *JAMA Health Forum.*

eTable 7. Changes in Clinical Outcomes by fracture/no fracture subgroup

	BPCI			Matched Controls			Diff-in-Diff Estimate		
	Baseline	Intervention	Diff	Baseline	Intervention	Diff	DID	Lower 95% CI	Upper 95% CI
**MJRLE with Fracture**									
**Readm: 30 Day**	11.5%	11.3%	-0.2%	11.1%	11.5%	0.3%	-0.5%	-3.8%	2.8%
**Readm: 90 Day**	20.7%	20.1%	-0.6%	19.2%	19.5%	0.3%	-0.9%	-4.9%	3.2%
**Mortality: 30 Day**	4.5%	2.7%	-1.8%	4.4%	5.0%	0.5%	-2.3%	-4.1%	-0.6%
**Mortality: 90 Day**	9.1%	7.2%	-1.9%	8.6%	9.0%	0.5%	-2.4%	-4.9%	0.1%
**ED visits: 30 Day**	15.0%	20.5%	5.5%	21.7%	20.7%	-1.0%	6.5%	-2.9%	15.9%
**ED visits: 90 Day**	24.9%	29.1%	4.3%	29.1%	30.3%	1.2%	3.1%	-7.3%	13.4%
**Healthy Days at Home**	66.9	70.7	3.9	66.5	68.6	2.0	1.8	-0.1	3.7
**% Discharged home**	5.3%	9.0%	3.8%	4.9%	7.1%	2.1%	1.7%	-1.0%	4.3%
**% with SNF stay**	72.2%	69.9%	-2.3%	67.5%	66.1%	-1.5%	-0.8%	-5.2%	3.6%
**% with HHA usage**	46.6%	48.3%	1.7%	49.2%	52.6%	3.4%	-1.6%	-6.0%	2.7%
**MJRLE with Fracture**									
**Readm: 30 Day**	4.1%	3.7%	-0.4%	4.1%	4.1%	-0.1%	-0.3%	-0.6%	0.0%
**Readm: 90 Day**	8.4%	7.3%	-1.1%	8.7%	8.5%	-0.1%	-1.0%	-1.4%	-0.5%
**Mortality: 30 Day**	0.2%	0.2%	0.0%	0.1%	0.2%	0.0%	0.0%	-0.1%	0.0%
**Mortality: 90 Day**	0.3%	0.3%	0.0%	0.3%	0.3%	0.0%	0.0%	-0.1%	0.0%
**ED visits: 30 Day**	6.9%	7.5%	0.6%	6.6%	7.3%	0.7%	-0.1%	-0.8%	0.7%
**ED visits: 90 Day**	11.7%	12.3%	0.6%	12.1%	12.6%	0.6%	0.0%	-0.9%	0.9%
**Healthy Days at Home**	83.3	85.0	1.8	83.5	84.6	1.1	0.6	0.4	0.8
**% Discharged home**	24.0%	44.0%	20.0%	22.6%	32.2%	9.7%	10.3%	6.3%	14.4%
**% with SNF stay**	33.0%	19.7%	-13.2%	29.7%	22.3%	-7.4%	-5.8%	-8.1%	-3.5%
**% with HHA usage**	58.9%	47.2%	-11.7%	62.8%	60.1%	-2.7%	-9.0%	-13.5%	-4.6%

BPCI=Bundled Payments for Care Improvement; DID=difference in differences; HHA=home health agency; MJRLE=major joint replacement of the lower extremity; SNF=skilled nursing facility. Outcomes are adjusted using patient-level comorbidities from Medicare's chronic conditions warehouse (CCW) data.

© 2021 Joynt Maddox et al. *JAMA Health Forum.*
